# Well-Differentiated Liposarcoma of the Posterior Neck: A Case Report

**DOI:** 10.7759/cureus.68179

**Published:** 2024-08-30

**Authors:** Nawaf Almotairi, Balqees Y Alghanem, Mohammad Alotaibi, Mishal M AlMutairi

**Affiliations:** 1 Otolaryngology, Head and Neck Surgery, Farwaniya Hospital, Al Farwaniyah, KWT; 2 Otolaryngology, Head and Neck Surgery, Kuwait Institute for Medical Specializations, Kuwait, KWT

**Keywords:** neck neoplasms, head, neck mass, soft tissue sarcoma, liposarcoma

## Abstract

Soft tissue sarcoma is a rare differential diagnosis for masses arising in the head and neck regions and should be evaluated carefully. We report a case of a patient with a large posterior neck mass. Initially, the mass was suspected to be an intermuscular lipoma based on computed tomography (CT) scan and magnetic resonance imaging (MRI) findings, which showed a large, well-defined, lobulated intermuscular fatty mass. The mass was excised through a cervical incision while preserving the surrounding vital muscular and neurovascular structures. Histopathological examination revealed a well-differentiated liposarcoma (WDLS) with adipocytes of various sizes, scattered nuclear atypia, and expanded fibrous septa containing atypical stromal spindle cells positive for MDM2 staining. These findings confirmed the mass as a WDLS. We describe our approach to the diagnosis and treatment of this condition in detail.

## Introduction

In soft tissue tumors, liposarcomas account for 5-6% of cases. They develop most commonly in the retroperitoneum, with only 5% of cases occurring in the head and neck [1]. Notably, for every malignant liposarcoma, there are approximately 120 benign lipomas [1]. Fifty to fifty-four years is the median age at diagnosis for sarcomas of the head and neck regions when children are considered, and 55 to 59 years when only adults are included. Most case series reveal a slightly higher prevalence of male patients compared to female patients [2].

Significant predictive markers for the overall survival and progression-free survival of patients with head and neck soft tissue sarcoma (STS) include age, tumor size, and grade, overall stage, and resection margin [3]. Clinical presentations also vary depending on the site of the primary tumor. Patients typically present with a painless neck mass and symptoms related to the tumor’s origin, such as nasal obstruction or epistaxis in sinonasal tumors, and cranial nerve deficits in skull base tumors [4]. This case report aims to describe the diagnosis and management of head and neck liposarcomas.

## Case presentation

A 68-year-old man presented to the Department of Otolaryngology clinic, Head and Neck Surgery, with a 15-year history of a huge mass on the right side of his neck extending to the upper back. The mass was slow-growing, painless, and did not cause breathing or swallowing problems. However, it restricted his neck’s range of motion, especially towards the right side and during extension. The patient denied symptoms such as fever, night sweats, weight loss, or neurological deficits. Additionally, he did not experience nausea, vomiting, redness, or drainage of the mass. His family history was negative for malignant and benign neoplasms, and there was no personal history of smoking or alcohol consumption.

**Figure 1:**
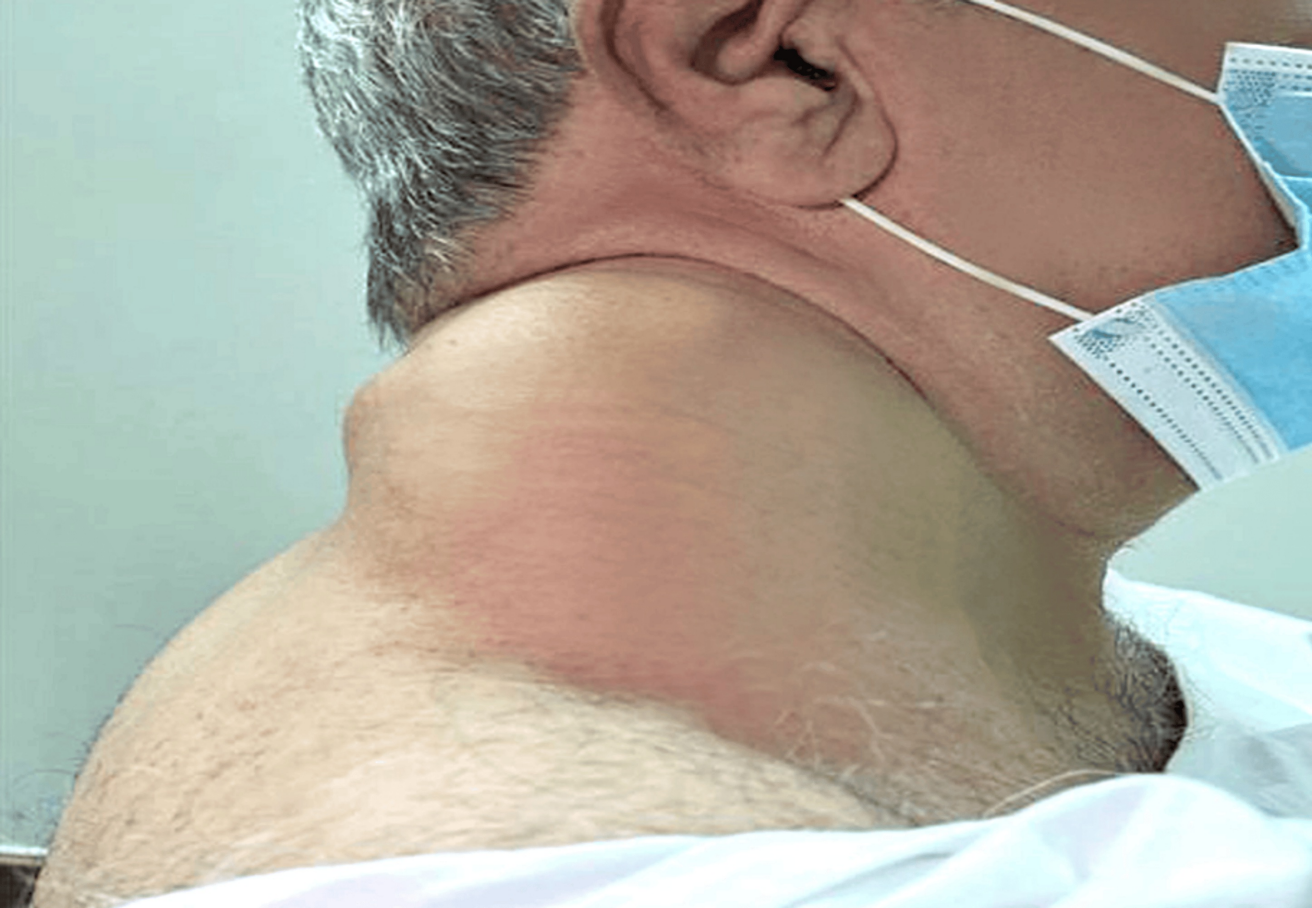
Lateral view of the neck showing a mass involving the posterior triangle.

**Figure 2:**
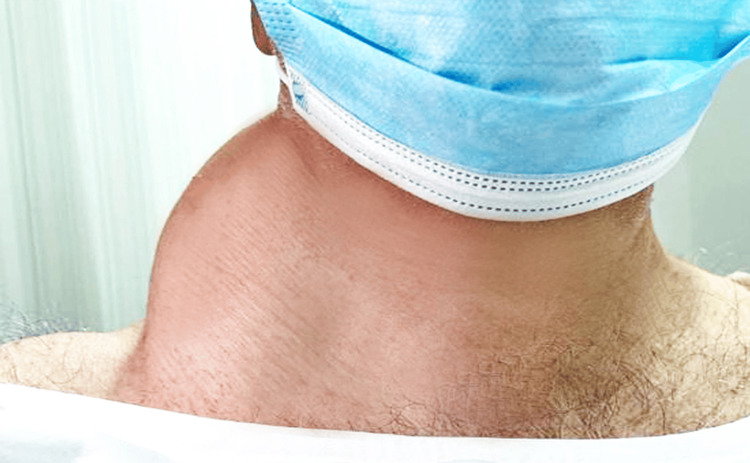
An anterior view of the neck shows a huge mass.

On physical examination, the patient was hemodynamically stable, with a BMI of 35. A focused neck assessment revealed a mass involving the right posterior triangle and extending to the upper aspects of the back. It measured approximately 14 cm × 24 cm × 20 cm in diameter. It was mobile with regular borders and a soft texture. Tenderness and changes in the overlying skin were not observed (Figures 1-2).

**Figure 3:**
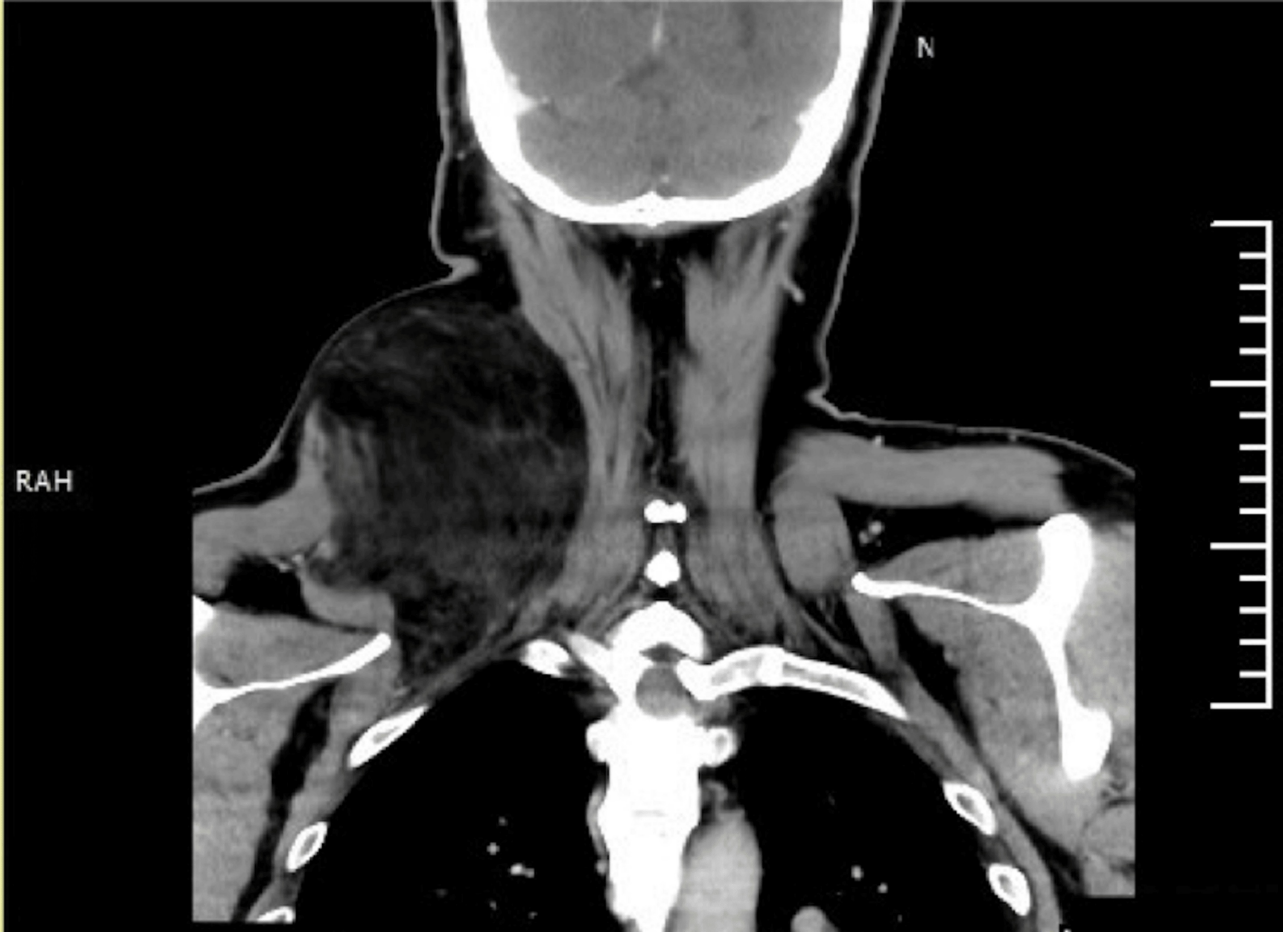
A coronal view of the neck CT scan shows a right-side mass compressing the surrounding muscular structures. The mass demonstrates multiple septations, small soft tissue nodules, fluid density areas, and curvilinear foci of calcifications, with no evidence of soft tissue enhancement.

**Figure 4:**
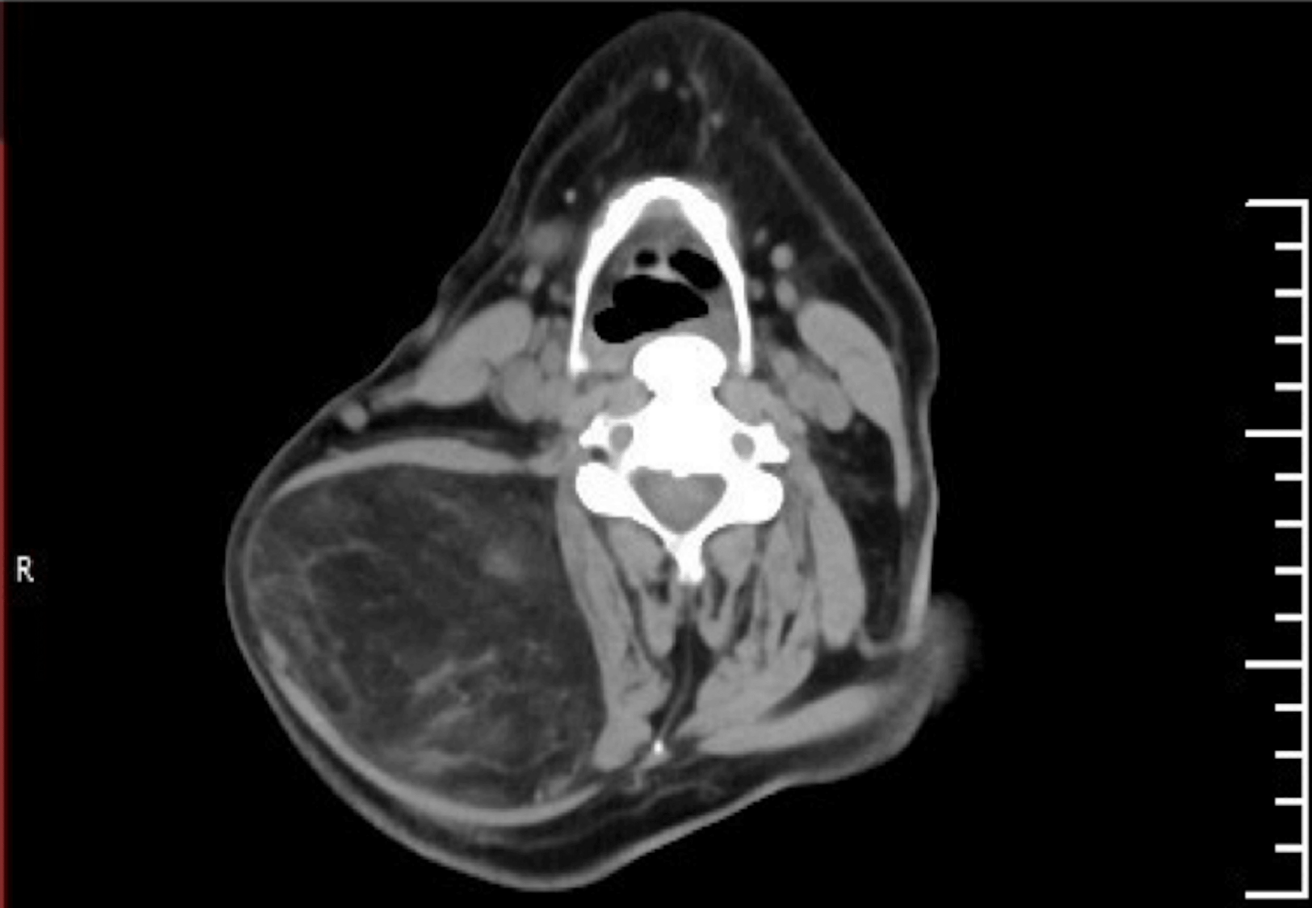
An axial view of the neck CT scan shows a huge right mass.

**Figure 5:**
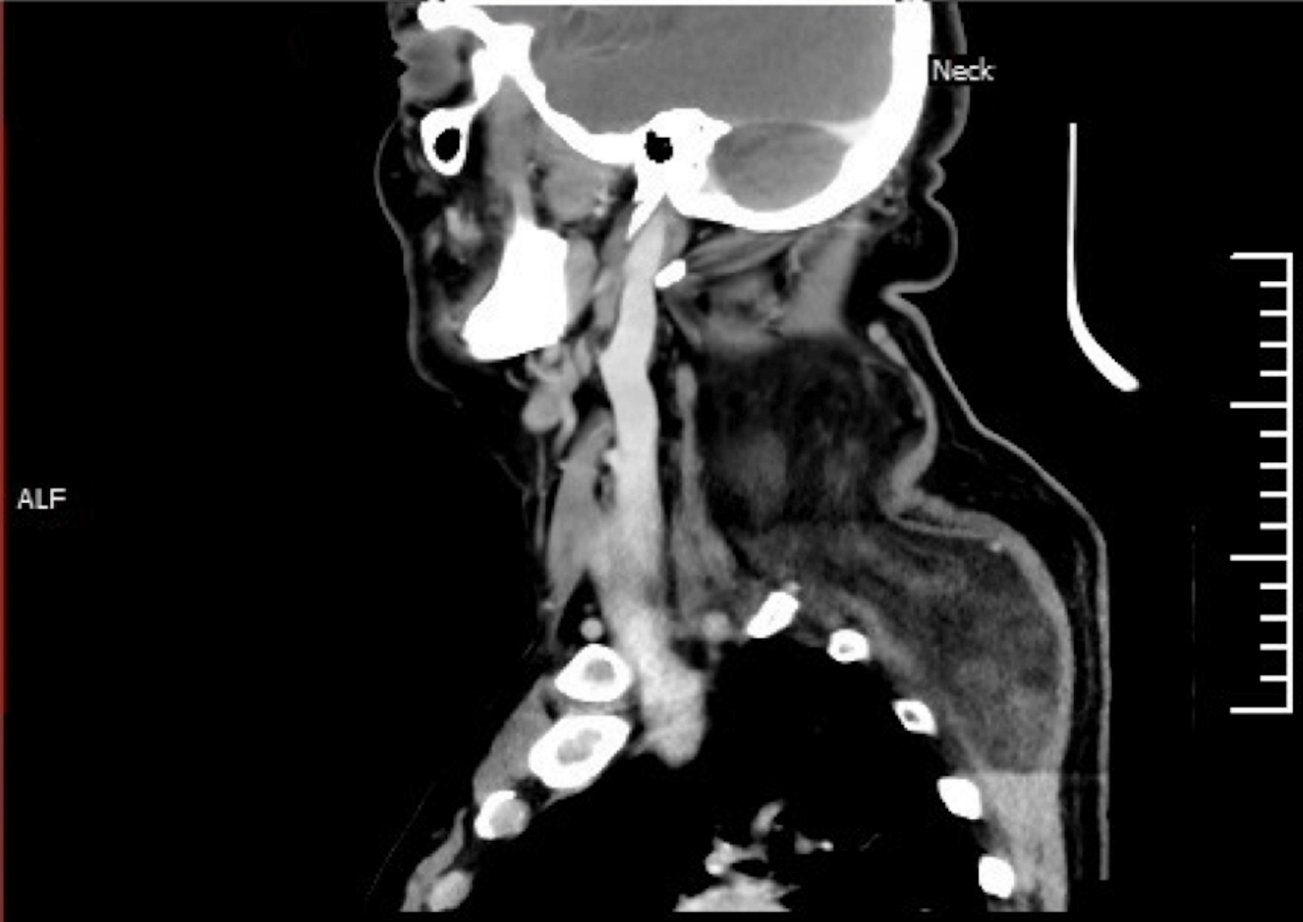
A sagittal view of the neck CT scan shows a huge cervical mass involving the posterior triangle and extending to the back.

The rest of the head and neck assessment was unremarkable. Three years prior to his presentation to our clinic, a neck ultrasound was conducted, revealing a mass measuring 8.1 cm x 4.6 cm below the subcutaneous fatty layer. Situated between the muscles, the lesion on the right posterolateral aspect of the neck was suggestive of a lipoma. Magnetic resonance imaging (MRI) of the neck revealed the presence of a large, well-defined, lobulated intermuscular fatty mass measuring approximately 16.9 x 9.45 x 11.3 cm, located within the posterolateral compartment of the neck and upper chest, further suggestive of an intermuscular lipoma. Contrast-enhanced computed tomography (CT) of the neck revealed a sizable mass, predominantly composed of fatty tissue, occupying the posterior paraspinal space with extension to the upper back. This mass had both inter- and intramuscular components involving the right splenius capitis and trapezius muscles, extending anteriorly into the right posterior triangle, and measured approximately 10.5 × 13.2 × 17 cm (Figures 3-5).

**Figure 6:**
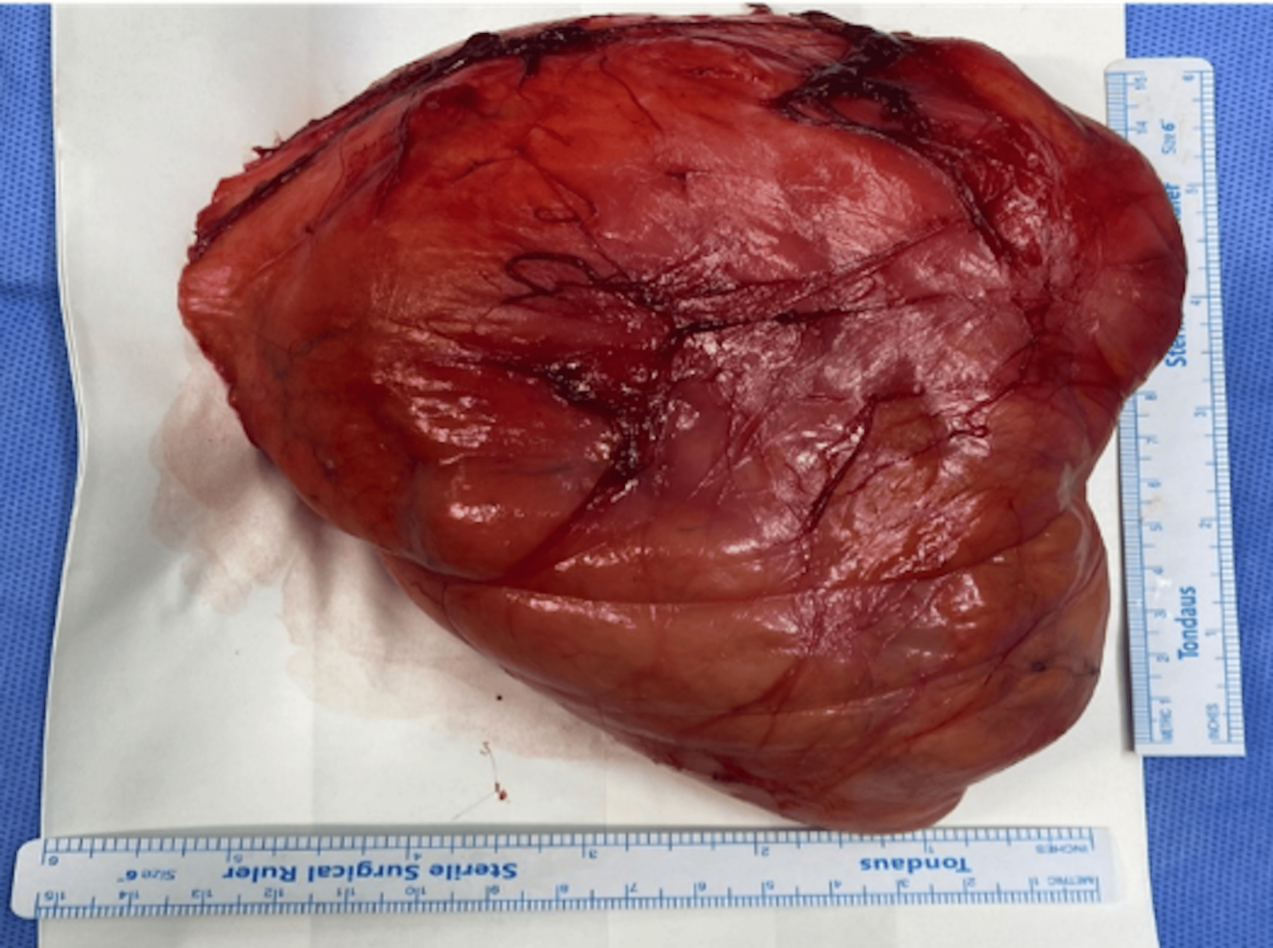
Gross appearance of the excised mass showing a well-circumscribed, oval-shaped lesion with a smooth surface and well-defined borders, measuring approximately 14 × 13.5 × 5.5 cm.

**Figure 7:**
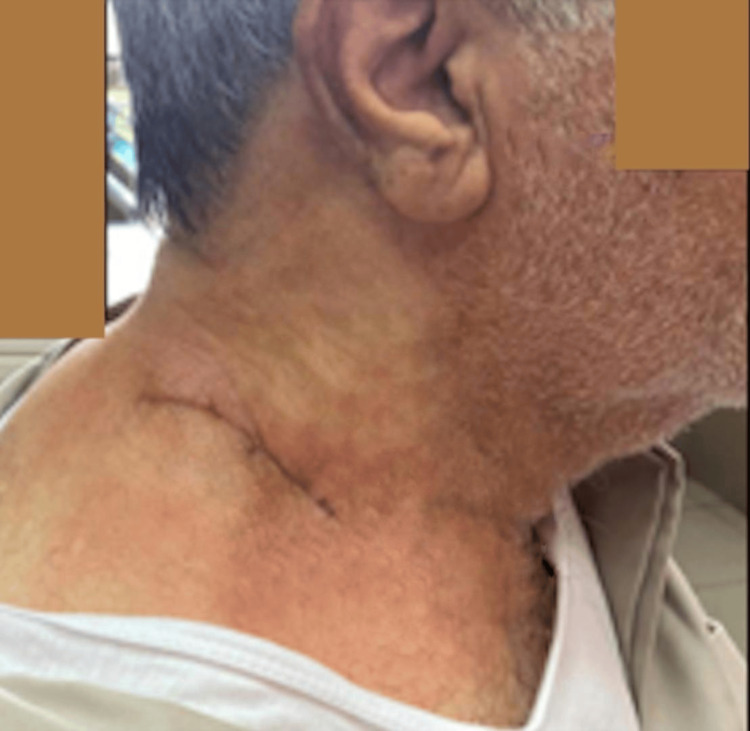
Postoperative appearance of the incision site showing a linear incision located on the posterior triangle of the neck. The incision is closed with sutures, with minimal surrounding erythema and no signs of infection. The edges of the incision are well-approximated, and the overall appearance suggests good healing progress.

Fine-needle aspiration cytology (FNAC) confirmed the presence of mature fibroadipose tissue consistent with a lipoma. The mass was excised under general anesthesia through a cervical incision while preserving the surrounding vital muscular and neurovascular structures (Figures 6-7).

**Figure 8:**
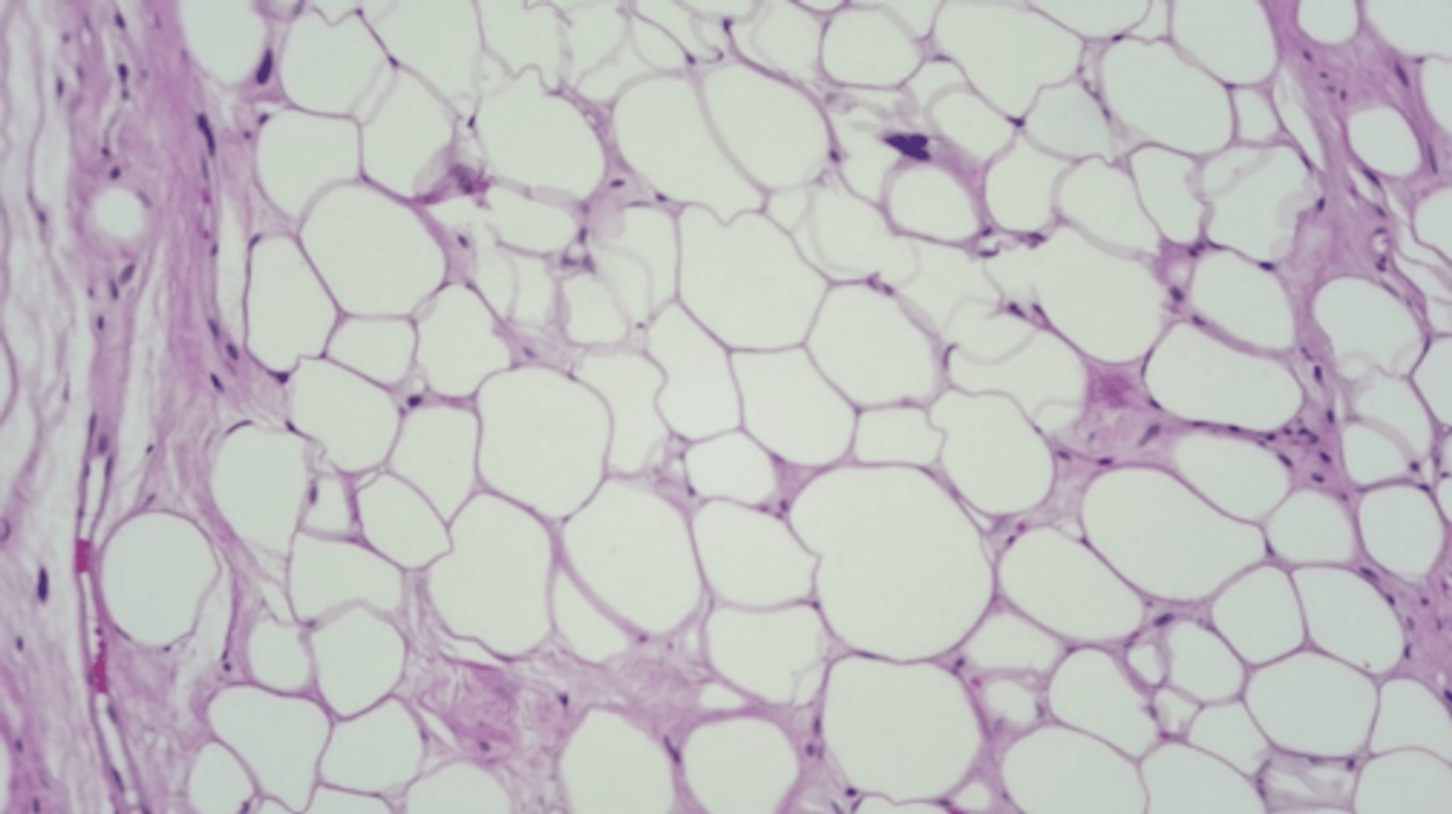
Atypical lipomatous tumor/well-differentiated liposarcoma showing adipocytes of various sizes and scattered nuclear atypia (hematoxylin and eosin stain, 100x).

**Figure 9:**
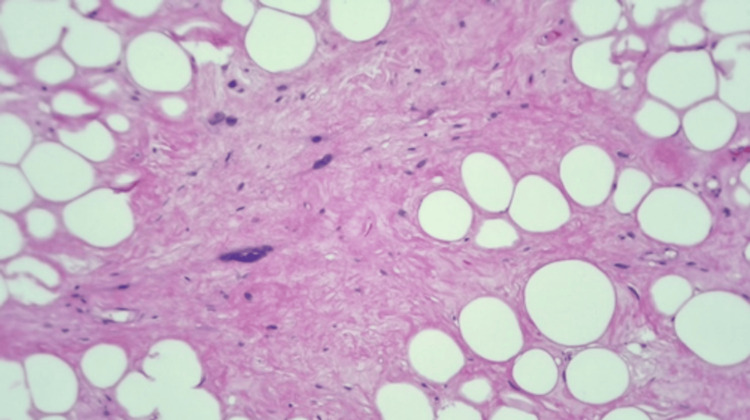
Atypical lipomatous tumor/well-differentiated liposarcoma with expanded fibrous septa containing atypical stromal spindle cells with enlarged hyperchromatic nuclei (hematoxylin and eosin stain, 100x).

**Figure 10:**
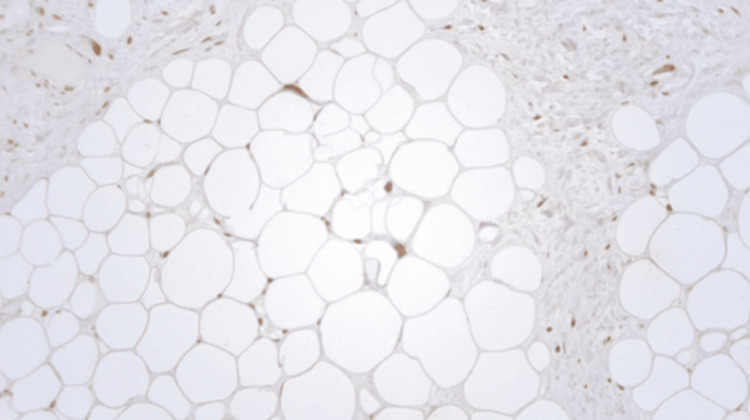
The adipocytic and atypical stromal spindle cells demonstrate strong nuclear positivity for MDM2 stain (MDM2 immunostain, 100x). MDM2: mouse double minute 2

Histopathological evaluation of the mass revealed a well-differentiated liposarcoma (WDLS) (14 × 13.5 × 5.5 cm) with adipocytes of various sizes and scattered nuclear atypia. It also showed expanded fibrous septa containing atypical stromal spindle cells with enlarged hyperchromatic nuclei that were positive for mouse double minute 2 (MDM2) immunohistochemical staining (Figures 8-10).

The patient recovered well postoperatively with no complications. At the six-month postoperative follow-up, the patient had no complaints.

## Discussion

A comprehensive examination of the head and neck, with a particular focus on the cranial nerves, is essential for all patients with a suspected head and neck neoplasm. MRI is the preferred neuroimaging study for most patients, often used alongside CT. This combination is highly beneficial for surgical planning [5].

Histopathological evaluation of the tumor plays a significant role in determining prognosis. Various subtypes of histopathology have been documented, including round cell, myxoid, well-differentiated, and pleomorphic lesions. Patients with well-differentiated and myxoid cells are reported to have a better prognosis and survival compared to those with pleomorphic and round cells, with a 50% mortality rate over a two-year follow-up period [6].

Distinguishing between lipoma and WDLS is essential, as it directly impacts the management plan. In a study by Asano et al., a scoring system was developed to accurately differentiate between WDLS and lipoma. This system combines clinical factors (such as age, tumor location, size, and depth), MRI findings (including septation thickness, contrast enhancement, and neurovascular involvement), and histopathological features (like nuclear atypia and the presence of lipoblasts). While fluorescence in situ hybridization (FISH) is the gold standard for differentiating WDLS from lipoma, its availability is limited in some medical centers. Therefore, this scoring system offers a more practical alternative that can be widely implemented [7].

In preoperative and postoperative care, the crucial role of a multidisciplinary team cannot be overemphasized. Surgical outcomes are significantly enhanced by the expert contributions from various medical specialties [8]. Surgery is considered the cornerstone of treatment for STS of the head and neck due to its proven efficacy and favorable outcomes. A study by O’steen et al., involving 34 patients, found that combining surgery with postoperative radiation therapy (RT) results in excellent disease outcomes for high-grade STS of the head and neck, as well as for those with close or positive margins [9].

Head and neck sarcomas are located close to critical structures and are limited by anatomical restrictions in the head and neck area. As a result, establishing the optimal wide resection margins for these tumors is typically difficult compared to the more attainable margins in other anatomical areas, such as the extremities. A study by de Bree et al., reviewing 41 adult patients treated for head and neck STS, found that surgical margins and lymph node metastases are key prognostic factors. The study also emphasized that obtaining negative margins in these tumors is particularly challenging due to the surrounding vital structures [10].

Chemotherapy, whether administered as adjuvant or neoadjuvant therapy, has not shown a significant improvement in local disease control or long-term survival, particularly for patients with high-grade STS of the head and neck [11]. Recognizing the molecular pathways underlying liposarcoma can enhance precise diagnosis and support personalized therapy planning. Molecular biology holds great promise for both diagnostic and therapeutic applications. The U.S. Food and Drug Administration is currently reviewing trabectedin, which appears to be sensitive to myxoid (round cell) liposarcomas. In well-differentiated and dedifferentiated liposarcomas, cyclin-dependent kinase 4 (CDK4) and MDM2 are overexpressed, providing promising targets for treatment studies [12].

## Conclusions

In managing head and neck sarcomas, a comprehensive examination, including a focus on cranial nerves and advanced imaging techniques such as MRI and CT, is crucial for accurate diagnosis and surgical planning. Histopathological evaluation plays a significant role in determining prognosis, with well-differentiated and myxoid cells indicating a better outcome than pleomorphic and round cells. A multidisciplinary team approach enhances surgical outcomes, with surgery remaining the cornerstone of treatment. While combining surgery with postoperative RT has shown favorable results, chemotherapy has not significantly improved local control or long-term survival for high-grade STS of the head and neck. Advances in molecular biology offer promising avenues for personalized therapy, with potential targets such as CDK4 and MDM2 in well-differentiated and dedifferentiated liposarcomas.

## References

[1] Casani AP, Marchetti M, Dallan I, Cagno MC, Berrettini S (2005). Liposarcoma of the cervico-nuchal region. Otolaryngol Head Neck Surg.

[2] Peng KA, Grogan T, Wang MB (2014). Head and neck sarcomas: analysis of the SEER database. Otolaryngol Head Neck Surg.

[3] Ku JY, Roh JL, Cho KJ, Song JS, Choi SH, Nam SY, Kim SY (2020). Risk factors for survival of head and neck soft tissue sarcomas: a comparison between 7th and 8th edition AJCC staging systems. Oral Oncol.

[4] Gil Z, Patel SG, Singh B (2007). Analysis of prognostic factors in 146 patients with anterior skull base sarcoma: an international collaborative study. Cancer.

[5] Sharon CE, Straker RJ 3rd, Karakousis GC (2022). The role of imaging in soft tissue sarcoma diagnosis and management. Surg Clin North Am.

[6] McCulloch TM, Makielski KH, McNutt MA (1992). Head and neck liposarcoma. A histopathologic reevaluation of reported cases. Arch Otolaryngol Head Neck Surg.

[7] Asano Y, Miwa S, Yamamoto N (2022). A scoring system combining clinical, radiological, and histopathological examinations for differential diagnosis between lipoma and atypical lipomatous tumor/well-differentiated liposarcoma. Sci Rep.

[8] Licitra L, Keilholz U, Tahara M (2016). Evaluation of the benefit and use of multidisciplinary teams in the treatment of head and neck cancer. Oral Oncol.

[9] O'steen L, Saldivar B, Kharod S, Bassett B, Morris CG, Mendenhall WM (2020). Radiotherapy for adult soft tissue sarcomas of the head and neck. Am J Clin Oncol.

[10] de Bree R, van der Valk P, Kuik DJ (2006). Prognostic factors in adult soft tissue sarcomas of the head and neck: a single-centre experience. Oral Oncol.

[11] Rapidis AD (2008). Sarcomas of the head and neck in adult patients: current concepts and future perspectives. Expert Rev Anticancer Ther.

[12] Abbas Manji G, Singer S, Koff A, Schwartz GK (2015). Application of molecular biology to individualize therapy for patients with liposarcoma. Am Soc Clin Oncol Educ Book.

